# 4,10-Diformyl-2,6,8,12-tetra­nitro-2,4,6,8,10,12-hexa­azatetra­cyclo­[5.5.0.0^5,9^.0^3,11^]dodeca­ne

**DOI:** 10.1107/S1600536809055676

**Published:** 2010-01-13

**Authors:** Huaxiong Chen, Rui Shi, Shusen Chen, Shaohua Jin, Lijie Li, Yanshan Shi

**Affiliations:** a School of Materials Science and Engineering, Beijing Institute of Technology, Beijing 100081, People’s Republic of China

## Abstract

The title compound TNDFIW, C_8_H_8_N_10_O_10_, is a caged heterocycle substituted with four nitro and two formyl groups. It is related to the hexa­azaisowurtzitane family of high-density high-energy polycyclic cage compounds. Four nitro groups are appended to the four N atoms of the two five-membered rings, while the other two formyl groups are attached to the two N atoms of the six-membered ring, which adopts a boat conformation. The compound has a cage structure which is constructed from one six-membered and two five-membered rings which are closed by a C—C bond, thus creating two seven-membered rings. There are a number of close intermolecular contacts [O⋯O = 2.827 (5), 2.853 (4) and 2.891 (5) Å; O⋯N = 2.746 (2) and 2.895 (2) Å] The calculated density of TNDFIW is 1.891 Mg m^−3^.

## Related literature

For the synthesis, structure and properties of a related compound, see: Keshavarz *et al.* (2009 ▶); Liu *et al.* (2006 ▶); Ou *et al.* (2000 ▶); Jin *et al.* (2009 ▶). For *sp* ^3^ bond angles, see: Zarychta *et al.* (2005 ▶).
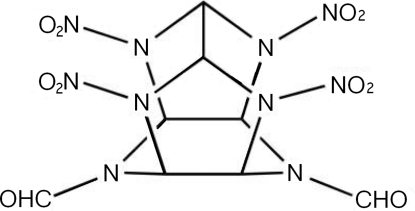


## Experimental

### 

#### Crystal data

C_8_H_8_N_10_O_10_
*M* *_r_* = 404.24
Orthorhombic, 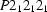

*a* = 8.7794 (15) Å
*b* = 12.715 (2) Å
*c* = 12.716 (2) Å
*V* = 1419.6 (4) Å^3^
*Z* = 4
Mo *K*α radiation
μ = 0.17 mm^−1^
*T* = 93 K
0.20 × 0.13 × 0.09 mm

#### Data collection

Rigaku AFC10/Saturn724+ diffractometer
11584 measured reflections
1866 independent reflections
1786 reflections with *I* > 2σ(*I*)
*R*_int_ = 0.047

#### Refinement

*R*[*F* ^2^ > 2σ(*F* ^2^)] = 0.041
*wR*(*F* ^2^) = 0.094
*S* = 1.00
1866 reflections
254 parameters
H-atom parameters constrained
Δρ_max_ = 0.25 e Å^−3^
Δρ_min_ = −0.22 e Å^−3^

### 

Data collection: *CrystalClear* (Rigaku, 2008 ▶); cell refinement: *CrystalClear*; data reduction: *CrystalClear*; program(s) used to solve structure: *SHELXS97* (Sheldrick, 2008 ▶); program(s) used to refine structure: *SHELXL97* (Sheldrick, 2008 ▶); molecular graphics: *SHELXTL* (Sheldrick, 2008 ▶); software used to prepare material for publication: *SHELXL97*.

## Supplementary Material

Crystal structure: contains datablocks I, global. DOI: 10.1107/S1600536809055676/fl2282sup1.cif

Structure factors: contains datablocks I. DOI: 10.1107/S1600536809055676/fl2282Isup2.hkl

Additional supplementary materials:  crystallographic information; 3D view; checkCIF report

## Figures and Tables

**Fig. 1.**
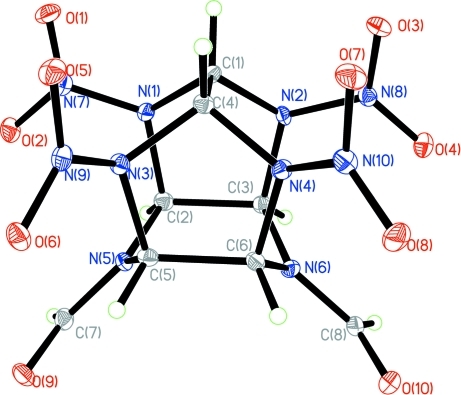
The structure of PTNDFIW, with displacement ellipsoids drawn at the 50% probability level.

## supplementary crystallographic information

### Comment

The title compound, TNDFIW, is an incompletely nitrated product of tetraacyl-diformyl-hexaazaisowurtzitane (TADFIW). Another incompletely-nitrated product of TADFIW, pentanitro-monoformyl-hexaazaisowurtzitane (PNMFIW), is a by-product formed in the synthesis of hexanitro-hexaazaisowurtzitane (HNIW) (Ou *et al.*, 2000; Liu *et al.*, 2006). The single-crystal structure of PNMFIW has been reported recently (Jin *et al.*, 2009). Slight variations of the structure of PNMFIW may result in a large decrease of sensitivity but with little loss of energy density according to the initiation mechanism (Keshavarz, *et al.*, 2009). Therefore, the HNIW derivatives substituted by fewer nitro groups such as PNMFIW and TNDFIW may have much lower sensitivity than HNIW but have similar energy.

The caged structure of HNIW is constructed from one six-membered and two five-membered rings which are closed by the C1—C4 bond, thus creating two seven-membered rings.The six-membered pyrazine ring has a boat conformation, while the more stable conformation of a six-membered ring is the chair form. Four nitro groups are appended to the four nitrogen atoms of the two five-membered rings, while two formyl groups are attached to the two nitrogen atoms of the six-membered ring. Due to caged structure of TNDFIW, the N—N (1.374–1.43 Å) bond length is much longer than that found in common nitramines (1.360 Å). The C—C bond lengths of TNDFIW (1.56–1.59 Å) are also much longer than normal C—C single bonds (1.54 Å). Bond angles in caged structures are also usually much larger than normal sp3 hybrid bond angles (Zarychta *et al.*, 2005). There are a number of close intermolecular contacts less than the van der Waals radii, such as O1···O3 (2.827 Å), O6···O8 (2.853 Å), O2···O4 (2.891 Å); O2···N9 (2.895 Å), and O8···N9 (2.746 Å). From the above analysis, we know that TNDFIW has high tensile force and energy.

### Experimental

Due to the higher electronic density of five-membered rings than six-membered rings, N atoms on the 2, 6, 8, 12-positions of the two five-membered rings are more reactive than the N atoms on the 8 and 10-positions of the six-membered ring. Therefore, the four acetyls on on the five-membered rings are cleaved first and replaced by nitro groups in the nitrolysis of TADFIW with mixed sulfuric and nitric acids as the nitrating agent.

Fuming sulfuric acid was slowly added into fuming nitric acid in a three-neck flask with stirring. After the solution of mixed acids was heated to 40 ^0^C, tetraacetyl-diformylhexaazaisowurtzitane (10 g) was added, and then the temperature was elevated to 45 ^0^C. The solution was maintained at 45 ^0^C for 8 h; thereafter the solution was poured into ice-water. The precipitated solid was filtered off, washed with water and then dried. The obtained solid was a mixture of polynitrohexaazaisowurtzitane derivatives with different numbers of nitro substitutes.

Pure TNDFIW was obtained using silica column chromatography with hexane/acetyl acetate (6/4 by volume) as the mobile phase at room temperature (25 ^0^C). Pure TNDFIW was dissolved in dry acetyl acetate, and then several drops of hexane was added. The resulting solution wasallowed to sit at ambient conditions (15–20 *^o^*C). A week later, single crystals was obtained by controlling the evaporation of the solvent. Element analysis, FT—IR, MS and ^1^H NMR were in agreement with the structure of TNDFIW.

### Refinement

All non-hydrogen atoms were obtained using the direct methods. The hydrogen atom were placed geometrically and treated by a constrained refinement. The distances of C1—H, C2—H,C3—H,C4—H,C5—H, C6—H are 1.000 A, and the distance of C7—H is 0.950 A. The *U*_eq_ of H is assigned 1.2 time *U*_eq_ of C linked. Since the absolute configuration for the compound could not reliably be determined from Mo Kα; data, the Friedel equivalents were merged before the final cycles of refinement.

### Crystal data

**Table:**

C_8_H_8_N_10_O_10_	*F*(000) = 824
*M**_r_* = 404.24	*D*_x_ = 1.891 Mg m^−^^3^
Orthorhombic, *P*2_1_2_1_2_1_	Mo *K*α radiation, λ = 0.71073 Å
Hall symbol: P 2ac 2ab	Cell parameters from 4366 reflections
*a* = 8.7794 (15) Å	θ = 3.2–27.5°
*b* = 12.715 (2) Å	µ = 0.17 mm^−^^1^
*c* = 12.716 (2) Å	*T* = 93 K
*V* = 1419.6 (4) Å^3^	Prism, colorless
*Z* = 4	0.20 × 0.13 × 0.09 mm

### Data collection

**Table:**

Rigaku AFC10/Saturn724+ diffractometer	1786 reflections with *I* > 2σ(*I*)
Radiation source: Rotating Anode	*R*_int_ = 0.047
graphite	θ_max_ = 27.5°, θ_min_ = 3.2°
Detector resolution: 28.5714 pixels mm^-1^	*h* = −11→11
Multi–scan	*k* = −16→15
11584 measured reflections	*l* = −16→16
1866 independent reflections	

### Refinement

**Table:**

Refinement on *F*^2^	Secondary atom site location: difference Fourier map
Least-squares matrix: full	Hydrogen site location: inferred from neighbouring sites
*R*[*F*^2^ > 2σ(*F*^2^)] = 0.041	H-atom parameters constrained
*wR*(*F*^2^) = 0.094	*w* = 1/[σ^2^(*F*_o_^2^) + (0.0526*P*)^2^ + 0.616*P*] where *P* = (*F*_o_^2^ + 2*F*_c_^2^)/3
*S* = 1.00	(Δ/σ)_max_ = 0.001
1866 reflections	Δρ_max_ = 0.25 e Å^−^^3^
254 parameters	Δρ_min_ = −0.22 e Å^−^^3^
0 restraints	Extinction correction: *SHELXL97* (Sheldrick, 2008), Fc^*^=kFc[1+0.001xFc^2^λ^3^/sin(2θ)]^-1/4^
Primary atom site location: structure-invariant direct methods	Extinction coefficient: 0.0059 (19)

### Special details

**Table:**

Geometry. All e.s.d.'s (except the e.s.d. in the dihedral angle between two l.s. planes) are estimated using the full covariance matrix. The cell e.s.d.'s are taken into account individually in the estimation of e.s.d.'s in distances, angles and torsion angles; correlations between e.s.d.'s in cell parameters are only used when they are defined by crystal symmetry. An approximate (isotropic) treatment of cell e.s.d.'s is used for estimating e.s.d.'s involving l.s. planes.
Refinement. Since the absolute configuration for the compound could not reliably be determined from Mo *K*α data, the Friedel equivalents were merged before the final cycles of refinement. Refinement of *F*^2^ against ALL reflections. The weighted *R*-factor *wR* and goodness of fit *S* are based on *F*^2^, conventional *R*-factors *R* are based on *F*, with *F* set to zero for negative *F*^2^. The threshold expression of *F*^2^ > σ(*F*^2^) is used only for calculating *R*-factors(gt) *etc*. and is not relevant to the choice of reflections for refinement. *R*-factors based on *F*^2^ are statistically about twice as large as those based on *F*, and *R*- factors based on ALL data will be even larger.

### Fractional atomic coordinates and isotropic or equivalent isotropic displacement parameters (Å^2^)

**Table:**

	*x*	*y*	*z*	*U*_iso_*/*U*_eq_	
O1	0.4082 (3)	0.85128 (17)	0.61717 (16)	0.0240 (5)	
O2	0.3469 (3)	0.90661 (16)	0.77458 (17)	0.0216 (5)	
O3	0.0964 (3)	0.53590 (17)	0.52348 (15)	0.0199 (5)	
O4	−0.0683 (2)	0.49964 (16)	0.64766 (17)	0.0211 (5)	
O5	0.6678 (2)	0.63739 (16)	0.65467 (17)	0.0210 (5)	
O6	0.6745 (2)	0.61863 (16)	0.82517 (17)	0.0209 (5)	
O7	0.4006 (3)	0.41001 (17)	0.57581 (16)	0.0233 (5)	
O8	0.3173 (3)	0.33820 (16)	0.72066 (18)	0.0256 (5)	
O9	0.4078 (2)	0.73460 (18)	1.03162 (16)	0.0227 (5)	
O10	0.0492 (3)	0.40004 (17)	0.91506 (17)	0.0239 (5)	
N1	0.2201 (3)	0.77417 (18)	0.70425 (19)	0.0156 (5)	
N2	0.0774 (3)	0.64219 (18)	0.66086 (18)	0.0143 (5)	
N3	0.4504 (3)	0.65013 (19)	0.74559 (18)	0.0149 (5)	
N4	0.2978 (3)	0.51090 (18)	0.70091 (18)	0.0143 (5)	
N5	0.2786 (3)	0.70758 (17)	0.87793 (18)	0.0142 (5)	
N6	0.1089 (3)	0.55208 (18)	0.82910 (18)	0.0149 (5)	
N7	0.3337 (3)	0.84824 (18)	0.69836 (19)	0.0177 (5)	
N8	0.0326 (3)	0.55254 (19)	0.60750 (19)	0.0163 (5)	
N9	0.6093 (3)	0.63184 (18)	0.7414 (2)	0.0164 (5)	
N10	0.3446 (3)	0.41296 (19)	0.66374 (19)	0.0179 (5)	
C1	0.2262 (3)	0.6855 (2)	0.6321 (2)	0.0152 (6)	
H1	0.2281	0.7093	0.5572	0.018*	
C2	0.1599 (3)	0.7422 (2)	0.8080 (2)	0.0166 (6)	
H2	0.0984	0.7998	0.8404	0.020*	
C3	0.0559 (3)	0.6462 (2)	0.7767 (2)	0.0147 (6)	
H3	−0.0530	0.6606	0.7947	0.018*	
C4	0.3630 (3)	0.6075 (2)	0.6572 (2)	0.0148 (6)	
H4	0.4281	0.5937	0.5942	0.018*	
C5	0.3705 (3)	0.6206 (2)	0.8431 (2)	0.0146 (6)	
H5	0.4451	0.5999	0.8989	0.018*	
C6	0.2672 (3)	0.5255 (2)	0.8130 (2)	0.0148 (6)	
H6	0.2958	0.4613	0.8540	0.018*	
C7	0.3069 (4)	0.7593 (2)	0.9714 (2)	0.0190 (6)	
H7	0.2439	0.8173	0.9895	0.023*	
C8	0.0109 (4)	0.4835 (2)	0.8771 (2)	0.0195 (6)	
H8	−0.0937	0.5025	0.8810	0.023*	

### Atomic displacement parameters (Å^2^)

**Table:**

	*U*^11^	*U*^22^	*U*^33^	*U*^12^	*U*^13^	*U*^23^
O1	0.0279 (12)	0.0256 (11)	0.0184 (10)	−0.0046 (10)	0.0081 (9)	0.0027 (9)
O2	0.0257 (12)	0.0141 (9)	0.0251 (11)	−0.0016 (9)	−0.0003 (9)	−0.0051 (9)
O3	0.0234 (12)	0.0236 (10)	0.0127 (9)	0.0024 (9)	0.0006 (9)	−0.0024 (8)
O4	0.0173 (11)	0.0193 (10)	0.0266 (11)	−0.0033 (9)	0.0003 (9)	−0.0016 (9)
O5	0.0170 (11)	0.0245 (11)	0.0217 (11)	0.0005 (9)	0.0064 (9)	−0.0007 (9)
O6	0.0191 (11)	0.0209 (10)	0.0226 (11)	0.0008 (9)	−0.0067 (9)	−0.0013 (8)
O7	0.0289 (12)	0.0235 (11)	0.0175 (10)	0.0060 (11)	0.0033 (9)	−0.0041 (9)
O8	0.0360 (14)	0.0130 (10)	0.0278 (12)	0.0012 (10)	0.0008 (11)	0.0026 (9)
O9	0.0232 (11)	0.0282 (11)	0.0167 (10)	−0.0023 (10)	−0.0049 (9)	−0.0026 (9)
O10	0.0285 (12)	0.0212 (11)	0.0221 (11)	−0.0076 (10)	−0.0001 (10)	0.0030 (9)
N1	0.0186 (12)	0.0131 (10)	0.0151 (11)	−0.0028 (10)	0.0005 (10)	−0.0001 (9)
N2	0.0156 (12)	0.0135 (11)	0.0136 (11)	−0.0018 (10)	−0.0010 (10)	−0.0035 (9)
N3	0.0118 (12)	0.0176 (11)	0.0152 (11)	0.0000 (10)	−0.0005 (10)	−0.0005 (9)
N4	0.0164 (12)	0.0116 (10)	0.0148 (11)	0.0008 (10)	0.0020 (9)	−0.0007 (9)
N5	0.0161 (12)	0.0137 (11)	0.0129 (11)	−0.0010 (9)	−0.0006 (10)	−0.0015 (9)
N6	0.0158 (12)	0.0142 (11)	0.0146 (11)	−0.0004 (9)	−0.0015 (10)	0.0025 (9)
N7	0.0169 (13)	0.0144 (11)	0.0218 (12)	0.0020 (10)	−0.0007 (11)	0.0021 (10)
N8	0.0176 (12)	0.0152 (11)	0.0161 (11)	0.0004 (10)	−0.0039 (10)	−0.0001 (9)
N9	0.0146 (12)	0.0128 (11)	0.0217 (12)	−0.0002 (10)	0.0014 (10)	−0.0017 (10)
N10	0.0203 (13)	0.0150 (11)	0.0184 (12)	0.0043 (10)	−0.0023 (11)	−0.0015 (10)
C1	0.0178 (14)	0.0135 (12)	0.0144 (13)	−0.0016 (11)	0.0009 (11)	0.0014 (10)
C2	0.0199 (15)	0.0145 (13)	0.0154 (13)	0.0006 (12)	−0.0001 (12)	−0.0004 (11)
C3	0.0132 (13)	0.0162 (13)	0.0146 (12)	0.0006 (11)	−0.0011 (11)	0.0011 (11)
C4	0.0174 (14)	0.0135 (12)	0.0134 (13)	0.0018 (11)	0.0000 (11)	−0.0008 (11)
C5	0.0141 (14)	0.0182 (13)	0.0116 (12)	0.0002 (11)	0.0005 (11)	0.0007 (10)
C6	0.0147 (14)	0.0173 (13)	0.0125 (13)	−0.0005 (11)	−0.0018 (11)	−0.0002 (10)
C7	0.0218 (15)	0.0174 (13)	0.0178 (14)	−0.0035 (12)	0.0014 (12)	−0.0040 (12)
C8	0.0211 (15)	0.0217 (15)	0.0156 (13)	−0.0087 (13)	−0.0013 (12)	0.0005 (12)

### Geometric parameters (Å, °)

**Table:**

O1—N7	1.223 (3)	N4—C6	1.462 (4)
O2—N7	1.226 (3)	N4—C4	1.464 (3)
O3—N8	1.225 (3)	N5—C7	1.381 (4)
O4—N8	1.224 (3)	N5—C5	1.439 (3)
O5—N9	1.218 (3)	N5—C2	1.439 (4)
O6—N9	1.221 (3)	N6—C8	1.369 (4)
O7—N10	1.222 (3)	N6—C6	1.445 (4)
O8—N10	1.219 (3)	N6—C3	1.446 (4)
O9—C7	1.212 (3)	C1—C4	1.590 (4)
O10—C8	1.213 (4)	C1—H1	1.0000
N1—N7	1.374 (3)	C2—C3	1.575 (4)
N1—C1	1.454 (3)	C2—H2	1.0000
N1—C2	1.478 (4)	C3—H3	1.0000
N2—N8	1.384 (3)	C4—H4	1.0000
N2—C1	1.464 (4)	C5—C6	1.559 (4)
N2—C3	1.486 (3)	C5—H5	1.0000
N3—N9	1.415 (3)	C6—H6	1.0000
N3—C4	1.465 (3)	C7—H7	0.9500
N3—C5	1.474 (3)	C8—H8	0.9500
N4—N10	1.394 (3)		
			
N7—N1—C1	118.1 (2)	N5—C2—N1	112.1 (2)
N7—N1—C2	119.8 (2)	N5—C2—C3	109.8 (2)
C1—N1—C2	111.3 (2)	N1—C2—C3	101.3 (2)
N8—N2—C1	116.2 (2)	N5—C2—H2	111.1
N8—N2—C3	118.6 (2)	N1—C2—H2	111.1
C1—N2—C3	110.4 (2)	C3—C2—H2	111.1
N9—N3—C4	115.3 (2)	N6—C3—N2	112.8 (2)
N9—N3—C5	117.3 (2)	N6—C3—C2	109.8 (2)
C4—N3—C5	107.6 (2)	N2—C3—C2	101.7 (2)
N10—N4—C6	119.9 (2)	N6—C3—H3	110.7
N10—N4—C4	120.4 (2)	N2—C3—H3	110.7
C6—N4—C4	109.6 (2)	C2—C3—H3	110.7
C7—N5—C5	122.0 (2)	N4—C4—N3	102.9 (2)
C7—N5—C2	121.1 (2)	N4—C4—C1	107.7 (2)
C5—N5—C2	116.8 (2)	N3—C4—C1	108.6 (2)
C8—N6—C6	121.3 (2)	N4—C4—H4	112.4
C8—N6—C3	122.0 (2)	N3—C4—H4	112.4
C6—N6—C3	116.0 (2)	C1—C4—H4	112.4
O1—N7—O2	126.7 (2)	N5—C5—N3	109.3 (2)
O1—N7—N1	117.1 (2)	N5—C5—C6	110.2 (2)
O2—N7—N1	116.1 (2)	N3—C5—C6	105.5 (2)
O4—N8—O3	126.9 (3)	N5—C5—H5	110.6
O4—N8—N2	117.0 (2)	N3—C5—H5	110.6
O3—N8—N2	116.1 (2)	C6—C5—H5	110.6
O5—N9—O6	126.9 (2)	N6—C6—N4	110.2 (2)
O5—N9—N3	116.1 (2)	N6—C6—C5	110.1 (2)
O6—N9—N3	116.8 (2)	N4—C6—C5	103.4 (2)
O8—N10—O7	126.8 (3)	N6—C6—H6	111.0
O8—N10—N4	115.9 (2)	N4—C6—H6	111.0
O7—N10—N4	117.1 (2)	C5—C6—H6	111.0
N1—C1—N2	95.8 (2)	O9—C7—N5	123.5 (3)
N1—C1—C4	112.6 (2)	O9—C7—H7	118.2
N2—C1—C4	112.9 (2)	N5—C7—H7	118.2
N1—C1—H1	111.5	O10—C8—N6	124.1 (3)
N2—C1—H1	111.5	O10—C8—H8	118.0
C4—C1—H1	111.5	N6—C8—H8	118.0
			
C1—N1—N7—O1	19.1 (3)	N5—C2—C3—N6	1.0 (3)
C2—N1—N7—O1	160.2 (3)	N1—C2—C3—N6	119.7 (2)
C1—N1—N7—O2	−162.8 (2)	N5—C2—C3—N2	−118.7 (2)
C2—N1—N7—O2	−21.7 (4)	N1—C2—C3—N2	0.0 (3)
C1—N2—N8—O4	161.1 (2)	N10—N4—C4—N3	−112.6 (3)
C3—N2—N8—O4	25.9 (4)	C6—N4—C4—N3	32.6 (3)
C1—N2—N8—O3	−20.4 (3)	N10—N4—C4—C1	132.8 (3)
C3—N2—N8—O3	−155.6 (2)	C6—N4—C4—C1	−82.0 (3)
C4—N3—N9—O5	38.0 (3)	N9—N3—C4—N4	100.9 (3)
C5—N3—N9—O5	166.3 (2)	C5—N3—C4—N4	−32.1 (3)
C4—N3—N9—O6	−146.3 (2)	N9—N3—C4—C1	−145.1 (2)
C5—N3—N9—O6	−18.0 (3)	C5—N3—C4—C1	81.9 (3)
C6—N4—N10—O8	19.8 (4)	N1—C1—C4—N4	108.5 (2)
C4—N4—N10—O8	161.4 (3)	N2—C1—C4—N4	1.3 (3)
C6—N4—N10—O7	−164.2 (3)	N1—C1—C4—N3	−2.3 (3)
C4—N4—N10—O7	−22.5 (4)	N2—C1—C4—N3	−109.5 (3)
N7—N1—C1—N2	−173.1 (2)	C7—N5—C5—N3	−115.4 (3)
C2—N1—C1—N2	42.6 (3)	C2—N5—C5—N3	61.5 (3)
N7—N1—C1—C4	69.1 (3)	C7—N5—C5—C6	129.1 (3)
C2—N1—C1—C4	−75.2 (3)	C2—N5—C5—C6	−54.0 (3)
N8—N2—C1—N1	179.2 (2)	N9—N3—C5—N5	130.0 (2)
C3—N2—C1—N1	−42.1 (3)	C4—N3—C5—N5	−98.1 (3)
N8—N2—C1—C4	−63.2 (3)	N9—N3—C5—C6	−111.5 (3)
C3—N2—C1—C4	75.4 (3)	C4—N3—C5—C6	20.4 (3)
C7—N5—C2—N1	118.5 (3)	C8—N6—C6—N4	111.2 (3)
C5—N5—C2—N1	−58.4 (3)	C3—N6—C6—N4	−58.9 (3)
C7—N5—C2—C3	−129.8 (3)	C8—N6—C6—C5	−135.4 (3)
C5—N5—C2—C3	53.4 (3)	C3—N6—C6—C5	54.4 (3)
N7—N1—C2—N5	−54.2 (3)	N10—N4—C6—N6	−117.0 (3)
C1—N1—C2—N5	89.3 (3)	C4—N4—C6—N6	97.6 (3)
N7—N1—C2—C3	−171.2 (2)	N10—N4—C6—C5	125.5 (3)
C1—N1—C2—C3	−27.7 (3)	C4—N4—C6—C5	−19.9 (3)
C8—N6—C3—N2	−112.2 (3)	N5—C5—C6—N6	−0.2 (3)
C6—N6—C3—N2	57.9 (3)	N3—C5—C6—N6	−118.1 (2)
C8—N6—C3—C2	135.2 (3)	N5—C5—C6—N4	117.4 (2)
C6—N6—C3—C2	−54.7 (3)	N3—C5—C6—N4	−0.4 (3)
N8—N2—C3—N6	47.3 (3)	C5—N5—C7—O9	−1.5 (4)
C1—N2—C3—N6	−90.3 (3)	C2—N5—C7—O9	−178.2 (3)
N8—N2—C3—C2	164.8 (2)	C6—N6—C8—O10	4.8 (4)
C1—N2—C3—C2	27.2 (3)	C3—N6—C8—O10	174.3 (3)
